# Effect of Trade Openness on Food Security in the EU: A Dynamic Panel Analysis

**DOI:** 10.3390/ijerph17124311

**Published:** 2020-06-16

**Authors:** Giulio Fusco, Benedetta Coluccia, Federica De Leo

**Affiliations:** Department of Economics and Management, University of Salento, 73100 Lecce LE, Italy; federica.deleo@unisalento.it

**Keywords:** food security, trade openness, Common Agricultural Policy (CAP), European countries, dynamic panel

## Abstract

The problem of food insecurity is growing across the world, including economically developed countries. In Europe, the question is not just about the total supply of foods, but it includes even the accessibility of prices and their nutritional and qualitative adequacy. In this context many countries recognize the importance of trade policies to ensure adequate levels of food security. The aim of this work was to analyze the impact of trade openness on the level of food security in European countries, using a dynamic panel analysis with the generalized method of moments (GMM) approach. We selected two different indicators of food security (average protein supply, average dietary energy supply adequacy) capable of offering information both on the quantity and on the nutritional quality of the food supply. In order to improve the robustness of the empirical results, we developed three different regressions, with three trade openness indicators (trade openness, tariff, globalization) for each food security indicator. The results showed that commercial opening has, on average, a statistically significant net positive impact on the food security of European countries. Additional results indicate that also economic development, together with the importance of the agricultural sector, can improve food security levels.

## 1. Introduction

Problems of food insecurity are growing across the world, including economically developed countries [1]. In Europe, around half a million people do not have regular and sufficient access to food and about 20 million families cannot regularly afford high quality meals (i.e., fish, meat, or their vegetarian equivalent) [2,3]. These numbers are expected to rise due to the Covid-19 pandemic.

According to the Food and Agriculture Organization (FAO) definition [4], food security is referred to as a condition in which all the people, at all times, have physical, social and economic access to sufficient, safe, and nutritious food to meet their dietary needs and food preferences for an active and healthy life.

This phenomenon has historically been a challenge confined primarily to the developing world [5], where, due to food insecurity, millions of people are still suffering from chronic undernourishment. In industrialized countries, food security has a wider dimension and includes economic access to food that people want to eat, without compromising other needs such as rent, fuel, debt repayments, etc. [6,7].

Therefore, even in Europe, concerns over food security have emerged due to the growing volatility of food prices since 2008, which was not followed by political measures aimed at adjusting wages and maintaining social welfare [8]. In this direction, the European problem is not just about the limited total supply of foods, but also the inaccessibility of prices, which makes some lack of food. However, global food reserves have also decreased significantly compared to the past, due to the continuous increase in population [9,10]. In addition to economic reasons, natural disasters (i.e., floods, droughts, earthquakes) have a negative impact on the stability of agricultural production, endangering the livelihood and food supply of millions of people around the world [11]. Over the past 50 years, the frequency and intensity of natural disasters associated with climate change have grown significantly. In particular, data show that the economic impact of a natural disaster on the agricultural sector has increased by about 20 times [12].

Moreover, the FAO [13] definition outlines the connection between food security and food safety, requiring that available and accessible foods are also healthy and nutritionally adequate. According to Carvalho [14], food safety is an indefectible component of food security, as the prevention and control of diseases of plants and animals contribute to favoring the constant availability of agricultural products over time. Attention to hygiene and health standards, moreover, could open up international commercial outlets and could favor the ability to purchase food, making it less expensive [15]. However, it should be noted that the sanitary and phytosanitary rules can also represent an obstacle to exports, especially for some less advanced countries, with a negative impact on their food security [16].

In this context, we should note that food security indirectly depends on the economic, sanitary, social, and political system, and could have direct consequences for human health. In this context economic globalization has a fundamental role in order to generate imbalances of wealth between countries; in fact the globalization process eliminated the boundaries to a large extent among countries and has raised the integration of economies in terms of goods, services, and capital flows, which are key factors in order to measure the food security level [17].

Therefore, it is one of the main objectives of the European Union, which counts it among the principal purposes of the Common Agricultural Policy (CAP). This policy provides economic aid to farmers to improve agricultural productivity and it ensures stable and inexpensive food supply [18]. In particular, it provides income support through direct payments to farmers who implement environmentally friendly agriculture, adopt market measures to cope with difficult economic times, and implement rural development measures. Moreover, in recent times, in order to achieve adequate levels of food security, many countries have recognized the importance of trade policies, developing reforms to reduce taxes on incoming goods and contributing to the growth of the international market aimed at eradicating poverty and improving the availability of food [19,20]. In particular, trade openness plays a crucial role in ensuring the continuity of supply, as it allows to produce products in the most suitable areas and to move them to countries with insufficient food supplies. In this way, supply and demand are smoothed out, price fluctuations are reduced, and each country can increase the quantity and the variety of products available to the national population, guaranteeing a good level of food security [21,22]. Besides, through imports, each country can decide to obtain the food resources that it needs at a lower cost than it would sustain by producing it domestically [21]. Moreover, according to Wacziarg and Welch [23], trade openness allows access to larger markets which give the opportunity to benefit from economies of scale, technological transfers, and knowledge spillovers.

Despite the relevance of the topic, studies analyzing the link between trade openness and food security are lacking and many of them have focused on developing countries, where hunger still persists [21,24,25]. An additional problem comes from the fact that previous studies largely used poverty indicators instead of direct food security indicators.

On the contrary, this study is part of an emerging literature that examines the problem of food security in economically developed countries such as Italy [26], Australia [5], United Kingdom [27], Ireland [28], and Canada [29]. Moreover, it contributes to another strand of literature on the analogies and differences between the European countries [30]. However, we considered the problem of food security in the entire European context for the first time, evaluating the impact of trade policies on both quantity and quality of supplies.

In particular, the aim of this work was to analyze the impact of trade openness on the level of food security in European countries (see Appendix A). Using two different indicators of food security, a dynamic panel analysis was adopted, which is suitable for assessing the effects of a long-term policy reform [21,31]. A dynamic panel analysis with the generalized method of moments (GMM) approach was employed to the account for unobserved heterogeneity and potential endogeneity of the explanatory variables. This approach enables us to take a broader perspective and to focus on the overall level of food security of the European population.

The paper is organized as follows: Section 2 explains the methodology applied in the empirical analysis; Section 3 describes the data used for the study; Section 4 presents and discusses the achieved results; Section 5 presents the main conclusions and the policy implications.

## 2. Materials and Methods

The aim of this work was to analyze the impact of trade openness on the level of food security in European countries. According to previous studies [21,32], in this work we adopt a dynamic model approach to examine the effects of trade openness on food security for a cross-section of countries.

In the analysis of the economic aspects, it becomes fundamental to analyze the dynamic aspect of the phenomenon, as the effects of economic policies could only be evident with the passage of time. According to previous studies, the suitable methodology for analyzing the dynamic effect of a phenomenon of time is the “dynamic panel”.

One simple way of allowing dynamic effects in panel data models is including a lagged dependent variable. It is well known that the introduction of the lagged dependent variable will generally mean that standard estimators are inconsistent. Consistent estimators can be found using the GMM estimator proposed by Arellano and Bond (1991). In the following lines all the passages are described.

This particular methodology allows to capture the dynamic aspects of the commercial reforms and to face the problem of the potential endogeneity that could derive from this specification. In fact, the continuous evolution of economic processes means that the effect of economic and trade policies is completely evident only in the long run [33]. Therefore, the dynamic model allows one to consider the effects of explanatory variables over time. Therefore, we considered current food safety levels as a function of previous levels and we built the following regression models:

(1) $$FS_{i,t}=a+\beta FS_{i,t-1}+\gamma TO_{i.t}+\delta CV_{i,t}+\mu_{i}+\lambda_{t}+\varepsilon_{i,t}i=1,\ldots 33,\;t=1,\ldots 18$$

where *FS*, which stands for food security, was our dependent variable that indicates the level of food security, in our analysis we used two different variables i.e., average protein supply and average dietary energy supply adequacy. Where average protein supply indicates national average protein expressed in grams per capita per day, while dietary energy supply adequacy is a percentage of the average dietary energy requirement in each country. We decided to use these indicators because they both offer a quantitative information on the caloric energy input of foods available for human consumption and a qualitative information on the nutritional value of foods, since the protein component represents the major macronutrient group [24,34]. *TO*, which stands for trade openness, was an independent variable that indicates the level of trade openness in each country. In this study, in order to test the robustness of the result, we chose to use three different indicators: trade openness [35], tariff [36], and globalization [37]. *CV* is a set of control variables used to determine the potential level of food safety in each country. Finally, $\mu_{i}$, e, and $\lambda_{t}$ are respectively countries fixed effects and time fixed effects, while $\varepsilon_{i,t}$ is the error term.

The use of the delayed dependent variable in the model causes the phenomenon called “dynamic panel bias” [38], because the lagged dependent variable is endogenous to the fixed effects in the error term, which leads to estimation problems. Normally, this estimation problem cannot be eliminated with fixed or random effects regressions, and the estimation with the ordinary least squares (OLS) method is distorted, because the lagged dependent variable is correlated with the error term $\varepsilon_{i,t}$.

The common approach to dealing with non-stationary data is to apply the difference operator in order to achieve a dynamic specification in raw differences.

(2) $$\Delta FS_{i,t}=a+\beta \Delta FS_{i,t-1}+\gamma \Delta TO_{i.t}+\delta \Delta CV_{i,t}+\mu_{i}+\lambda_{t}+\varepsilon_{i,t}i=1,\ldots 33,\;t=1,\ldots 18$$

However, this approach is capable of removing the potential distortion, as it eliminated individual effect, because it doesn’t remove the temporal effect.

In order to solve this problem Holtz-Eakin et al. in 1988 [39] and Arellano and Bond in 1991 [40] developed an estimator for linear dynamic panel data models, called the generalized method of moments (GMM).

Despite the superiority of the difference-GMM (first order condition and GMM) estimator over the simpler panel data estimations, if the series are very repeated the lagged levels have been demonstrated to be ineffective tools for first-differences [41]. Then the performance of the difference-GMM estimator can be distorted for the small sample [42].

According to Arellano-Bover [40] and Blundell-Bond [43] the estimator performance can be increased by adding the original equation in levels to the system, which is known as the ‘‘system-GMM”. The peculiarity of the system-GMM estimator is that it weighs the moments in inverse proportion respecting their variances and covariances, for this reason, it reduces the weight in the estimation process of the instruments highly correlated.

## 3. Data Description

We used panel data composed by the European countries over the period 2000–2017 for the dependent variable (average dietary energy supply adequacy). Regarding the dependent variable, average protein supply, we analyze the period 2000–2012 due to a lack of data for the remaining years.

The variables were selected based on the FAO [44] and through the analysis of the previous empirical literature [18,42,43]. Most part of the data used in this study can be extracted from world development indicators and Food and Agriculture Organization Corporate Statistical Database (FAOSTAT). Moreover, according to Dithmer and Abdulai [21], we considered four groups of food security determinants: the first group describes the general context of the country; the second group captures the economic and demographic development; in the third group there were control variables that measure domestic macroeconomic policies and conditions; finally in the last group we considered non-economic events such as natural disasters.

In regards the first group, we took into consideration the total amount of economic resources, the availability of resources for agricultural production, and the importance of agriculture [45].

In particular, we used the gross domestic product (GDP) per capita as the principle variable to measure the quantity of final goods and services on the territory of a country. Rural population shares the variable which indicates the importance of agriculture and refers to the share of people living in rural areas out of the total population. The availability of resources for agricultural production is measured by the arable land variable, which includes land under temporary crops, temporary meadows for mowing or for pasture, land under market or kitchen gardens, and land temporarily fallow.

With regard to the second group of food security determinants, the model included three different variables that capture the agricultural, economic, and demographic development.

In particular, in order to capture the agricultural development, we use Cereal yield (kg per hectare; FAOSTAT), as a proxy for agricultural productivity. The economic development is measured by the gross domestic product (GDP) growth rate per capita variable. Finally, the population growth variable captures the demographic development.

In regards the third group, the inflation variable, measured by the consumer price index inflation rate, expresses the domestic macroeconomic policy quality; in particular, according to Loayza et al. [46], high inflation being associated with bad macroeconomic policies.

In the four group we used natural disaster variables; this value indicates the intensity of natural disasters and it is computed through a ratio between the number of populations affected by natural disasters and the total population for each country.

Finally, regarding the last group, we selected three different variables of trade openness, in order to test the research question.

The trade openness variable, according to Heston et al. [35], is a ratio between trade (real export and import) and GDP. The second variable was tariff [36], which indicates ad-valorem tariff, measured as import duties. Globalization [37] was the last variable, whereby we used the KOF (KOF is an acronym for the German word “Konjunkturforschungsstelle”, meaning: “economic cycle research institute”.) globalization index (0–100); The KOF index attempts to measure the degree to which a nation exchanges goods, capital, people, ideas, and information. It is a composite index that uses three dimensions: economic, social, and political, where a value close to 100 indicates a high level of globalization.

Table 1 and Table 2 present the variables, source of data, and their summary statistics.

As previously mentioned, the aim of this study was to estimate the impact of the trade openness on food security through a dynamic panel model approach. According to prior studies [21,31], we used the econometric structure described in the Equation (1). In order to improve the robustness of the empirical results developed three regressions, with three different trade openness indicators (trade openness, tariff, and globalization).

(3) $$\begin{array}{cc} FS_{i,t}=a+\beta FS_{i,t-1} & +\gamma TradeOpenness_{i.t}+\delta LnGDPpercapita_{i,t}\\ & +\vartheta GDPgrowth_{i,t}+\theta LnArableland_{i,t}\\ & +\pi lnAgriculturalproduction_{i,t}+{\rho \mathrm{Ruralpop}}_{i,t}\\ & +{\varphi \mathrm{LnPopgrowth}}_{i,t}+\tau Naturaldisaster_{i,t}\\ & +{\;\omega \mathrm{LnInflation}}_{i,t}+{\;\mu }_{i}+\lambda_{t}+\varepsilon_{i,t} \end{array}$$

(4) $$\begin{array}{cc} FS_{i,t}=a+\beta FS_{i,t-1} & +\gamma Tariff_{i.t}+\delta LnGDPpercapita_{i,t}+\vartheta GDPgrowth_{i,t}\\ & +\theta LnArableland_{i,t}+\pi lnAgriculturalproduction_{i,t}\\ & +{\rho \mathrm{Ruralpop}}_{i,t}+{\varphi \mathrm{LnPopgrowth}}_{i,t}\\ & +\tau Naturaldisaster_{i,t}+{\omega \mathrm{LnInflation}}_{i,t}+{\;\mu }_{i}+\lambda_{t}+\varepsilon_{i,t} \end{array}$$

(5) $$\begin{array}{cc} FS_{i,t}=a+\beta FS_{i,t-1} & +\gamma Globalization_{i.t}+\delta LnGDPpercapita_{i,t}\\ & +\vartheta GDPgrowth_{i,t}+\theta LnArableland_{i,t}\\ & +\pi lnAgriculturalproduction_{i,t}+{\rho \mathrm{Ruralpop}}_{i,t}\\ & +{\varphi \mathrm{LnPopgrowth}}_{i,t}+\tau Naturaldisaster_{i,t}\\ & +{\omega \mathrm{LnInflation}}_{i,t}+{\;\mu }_{i}+\lambda_{t}+\varepsilon_{i,t} \end{array}$$

## 4. Results and Discussions

**Figure 1:**
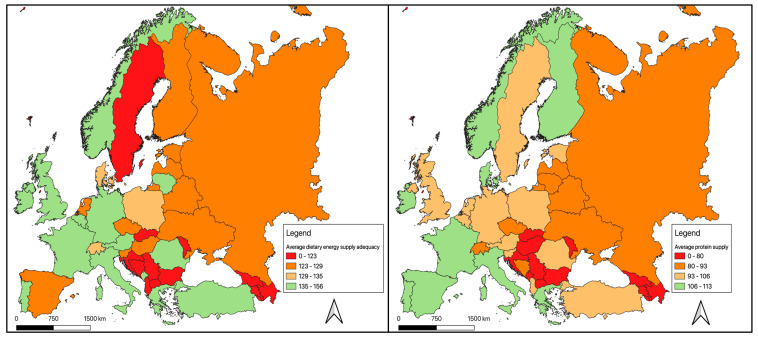
Geographical distribution of food security level in European countries.

In Europe, the levels of food security, assessed through the average dietary energy supply adequacy and the average protein supply variables, were lower in Eastern area (Figure 1). In particular, from an initial exploratory analysis, it emerged that the countries characterized by greater economic development were also characterized by a certain stability in the food supply.

Considering the possibility of collinearity in the model, we computed a correlation analysis between the independent variables. The correlation results are summarized in Table 3 [47]. From the results of the correlation analysis, we can affirm that there was no collinearity in the model, and for this reason we could preserve the regression model.

Table 4 shows the results of the three separate dynamic panel model regressions. The values in the table are coefficients, their *p*-value, the standard errors (in parentheses), and summary statistics.

The first column of Table 4 shows the relationship between our dependent variable (average protein supply) and the independent variables. In the first model we used Trade Openness (TO) as a variable to represent the level of commercial openness for each country. The lagged dependent variable was strongly significant, indicating that the level of food security changes only slowly over time and it depends on the past levels. This result also justifies the dynamic model specification and the employment of the system-GMM approach. From the results, we can affirm, according to previous studies [21,45], that when the volume of trade increases, a country’s food security level has prospects for improvement. According to previous studies, the increase of food supply should generate a reduction in consumer prices, facilitating the purchase of food products, in particular for developed countries [44].

In addition, our empirical results highlighted the importance of the economic aspect on food security level; the coefficient between our dependent variable (average protein supply) and the independent variable (GDP per capita) was positive and significant. Therefore, in countries with higher incomes, citizens have access to good quality food [48]. However, this value should not be understood simply as purchasing power, but also as the ability to adopt better technologies and to improve the level of food security [49]. The impact of the primary sector on food security was confirmed by our results, in fact, the relationship between average protein supply and arable land was positive and significant, and the same result was valid for the independent variable of rural population. In particular, the coefficient of arable land captures an important aspect of domestic resource endowments, and its value indicates that the households with larger arable land having a higher level of production are more likely to be food secure [49]. However, one of the weak points of the European agricultural sector is represented by the growing impermeabilization and the constant loss of soil productivity. It has been estimated that 18% of the cultivated land undergoes a decrease in productivity every year and that urbanized areas have grown by 78% in the last 50 years, increasingly limiting areas for cultivation [50]. The phenomenon is particularly felt in the area overlooking the Black Sea, but the Mediterranean regions are also very affected, due to intensive land use and expansion of urbanized areas. In the long run, this phenomenon could negatively affect the food security levels [51].

Finally, the value of the coefficient of the inflation variable was positive and significant, showing the importance of the macroeconomic policies in ensuring good levels of food security.

The column labelled (2) and (3) shows the results of the additional analysis to assess the robustness of our empirical results, in fact, we use alternative trade openness measures as tariff and globalization.

As shown in column (2), the ad-valorem tariff measure was significantly negatively related to average protein supply, implying that trade restrictions, on average, have detrimental effects on overall food security level. The relationship with the other independent variables was the same of the column (1).

Column (3) shows the results with the globalization variable, demonstrating that the relationship between globalization and the average protein supply variable was not significant between the European countries.

The column labelled (4) of Table 4 shows the analysis carried out with an alternative food security indicator, in particular it describes the relationship between the dependent variable (average dietary energy supply adequacy) and the other selected independent variables. In column (4) we used trade openness as the variable that expresses the level of commercial openness. Also in this case, the lagged dependent variable was strongly significant. From an empirical point of view, according to previous studies [21,44], when the volume of trade increases, in general thanks to a trade liberalization policy, food security levels also have the potential to improve. In fact, policies aimed at encouraging trade allow countries to access the global market, increasing the overall quantity of food and raw materials for agriculture [20]. Indeed, each state can use export earnings to import at an affordable price all those goods whose domestic production is scarce or too expensive [23]. Furthermore, according to Jaffe et al. [52], commercial opening is beneficial to food security levels because it can positively affect the employment of citizens, especially in the case of the least developed countries. The latter, thanks to the trade openness, can import products made with the relatively abundant factor and with low-skilled labor, thereby creating employment opportunities and raising workers’ incomes [48].

In addition, this alternative indicator also confirms the importance of the economic and agricultural aspects to guarantee good levels of food security. Indeed, the relationship between the dependent variable (average Energy Supply Adequacy) and the dependent variables (GDP pro capita, Arable Land, Rural Population, and Coefficient of Agricultural production) was positive and significant.

In addition, the empirical result confirms the importance of the non-economic evidence for the food security levels: the relationship between the average energy supply adequacy and the natural disaster independent variable was positive and significant, in particular, when the number of natural disaster decreases, the level of food security increases.

Moreover, from the results showed in the column labeled (5), we can confirm that the decrease in customs duties has a positive impact on improving food security levels.

Finally, as shown in the column (6), the relationship between globalization and our food security indicator was not significant in European countries.

## 5. Conclusions and Policy Implications

In the present study, through the use of a dynamic modeling approach, we have shown that commercial opening has, on average, a statistically significant net positive impact on the food security of European countries both from an energy and nutritional point of view.

This implies that commercial openness, in an economically advanced context, can have a positive impact both on security of supply, but also on the nutritional quality of the same, demonstrating the effectiveness of the commercial model proposed by the European Union, where the food sector represents a key resource from an economic, social and cultural point of view. In addition, our analysis confirmed that the most resilient countries are those characterized by higher per capita incomes.

Furthermore, the results showed that economic development, together with the importance of the agricultural sector in terms of production level, extension of agricultural land, and percentage of inhabitants in rural areas, have a significant positive impact. From the results of the analysis it is evident the effect of the single market established by the European Union (EU), in fact, the EU trade reform has been producing advantages in terms of increasing the level of food security. Moreover, the policies implemented by the EU on the agriculture field can also have an important impact on the quantity and quality of food supplies. The last reform was the CAP (2014–2020), which aims to help farmers to produce enough food for Europe, ensuring safety and quality at affordable prices. The objectives of the CAP aim to ensure a fair standard of living for farmers, protecting them from excessive volatility of prices, market crises, and imbalances within the food supply chain [18,53]. However, in order to continue guaranteeing adequate quantities of food in the long run, considering the continuous growth of the European population and the continuous pressures that agriculture exerts on the environment, it is necessary to act by promoting sustainable agricultural practices, that meet the current demand for food without compromising the ability of future generations to meet their needs [54].

Moreover, based on the results obtained from this study, in order to ensure quantity and quality of food supplies, it would be desirable for the European Union to adopt a liberal trade policy, which should represent a complement and not a substitute for domestic development policies. In addition, our analysis revealed that natural disasters also have a negative effect on food security levels. Agriculture, in fact, is a sector particularly exposed to natural risks (i.e., droughts, floods, earthquakes), which cause big economic losses every year. The growing frequency and complexity of these phenomena, and the projection that climate change will lead to an increase in extreme weather events [55], making it necessary to implement prevention policies that deal with agricultural losses and that ensure food supply flows [56]. In this context, it becomes crucial to put in place public policies aimed at ensuring the spread of the insurance instrument, in order to increase farmers’ productivity, limit damage from natural disasters, and strengthen resilience to food and nutritional insecurity [57,58].

Although this document has assessed and confirmed the advantages deriving from the trade openness, it is essential to specify that they could generate significant negative externalities. Currently, due to the globalization process, the seed market is controlled by just a handful of corporations, with damage in terms of biodiversity and healthiness of production, as well as negative economic consequences for small farmers [59]. In this context, CAP plays a key role thanks to the disbursement of funds that ensure income stability for 22 million small European farmers, which provide a large variety of accessible, safe, and good quality products [18].

Further research is needed to explore how the food security discourse is likely to evolve in the future, considering more indicators and increasing the analysis sample.

## Figures and Tables

**Table 1:** Variables and data sources (FAOSTAT stands for Food and Agriculture Organization Corporate Statistical Database; GDP stands for gross domestic product; EM-DAT stands for Emergency Events Database).

Variables	Unit	Data Source	Time Period
Average protein supply	(g/cap/day)	FAOSTAT	2000–2012
Average dietary energy supply adequacy	Percentage %	FAOSTAT	2000–2017
Trade openness	N°	World Development Indicators	2000–2017
Tariff	N°	World Development Indicators	2000–2017
Globalization	N°	Swiss Economic Institute	2000–2017
GDP per capita	US$	World Development Indicators	2000–2017
GDP growth	Percentage %	World Development Indicators	2000–2017
Arable land	(hectares per person)	FAOSTAT	2000–2017
Agricultural Production	(kg per hectare)	World Development Indicators	2000–2017
Rural population	Percentage %	World Development Indicators	2000–2017
Population growth	Annual Percentage %	World Development Indicators	2000–2017
Natural disaster	N°	EM-DAT	2000–2017
Inflation	Annual Percentage %	World Development Indicators	2000–2017

**Table 2:** Summary Statistics.

Variables	Mean	Standard Deviation	Maximum	Minimum
Average protein supply	97.780	12.399	118	67
Average dietary energy supply adequacy	129.304%	11.546%	158%	101%
Trade openness	1.009	0.503	3.785	0.408
Tariff	3.20	1.777	11.9	1.22
Globalization	78.6	9.382	91.313	47.509
GDP per capita	27,028.053	2,3952.970	118,823.648	354.003
GDP growth	2.734%	3.078%	25.162%	−14.758%
Arable land	8,741,911.189	21,322,651.81	124,374,000	60,000
Agricultural Production	2,227,506,805	3,535,306,006	21,419,375,209	1,010,275
Rural population	30.08%	13.053%	58.259%	2.039%
Population growth	0.225%	0.802%	2.890%	−3.847%
Natural Disaster	0.0049	0.0224	0.442	0
Inflation	4.598%	10.085%	168.620%	−4.478%

**Table 3:** Correlation analysis.

Variables	Trade Op.	Tariff	Glob	GDP Percap	GDP Growth	Arable Land	Agr. Prod.	Rur. Pop.	Pop. Growth	Nat. Dis.	Inf.
Trade openness	1	0.116	0.167	0.393	0.119	−0.271	−0.365	−0.139	0.241	0.001	−0.035
Tariff		1	−0.579	−0.276	−0.248	0.497	0.089	0.166	−0.205	−0.121	0.225
Glob			1	0.708	−0.335	−0.226	0.007	−0.624	0.422	−0.168	−0.462
GDPpercap				1	−0.203	−0.213	−0.090	−0.601	0.637	−0.080	−0.274
GDPgrowth					1	0.054	−0.067	0.192	−0.073	−0.002	0.102
Arable land						1	0.612	−0.065	−0.079	−0.023	0.190
Agr.Prod.							1	−0.123	0.030	−0.040	0.077
Rur.pop.								1	−0.399	0.133	0.093
Pop.growth									1	−0.061	−0.111
Nat.disaster										1	0.21
Inflation											1

Note: Trade op. stands for trade openness; Glob stands for globalization; GDPpercap stands for gross domestic product per capita; GDPgrowth stands for gross domestic product growth; Agr.Prod. stands for agricultural production; Rur.pop. stands for rural population; Nat.dis. stands for natural disasters; Inf. stands for inflation.

**Table 4:** Dynamic panel model regressions.

Variables	Av. Protein Supply (1)	Av. Protein Supply (2)	Av. Protein Supply (3)	Av. Dietary Energy Supply Adequacy (4)	Av. Dietary Energy Supply Adequacy (5)	Av. Dietary Energy Supply Adequacy (6)
Av. Protein supply adequacy t-1	0.06774250 *** (0.0163096)	0.0969006 *** (0.0146225)	0.0779083 *** (0.0144783)			
Av. Dietary energy supply adequacy t-1				0.944329 *** (0.0123442)	0.934857 *** (0.0126720)	0.941284*** (0.0122497)
Trade openness	0.0754607 *** (0.0142629)			0.293482 *** (0.104098)		
Tariff		−0.0123728 *** (0.00362975)			−0.0742521 ** (0.0325249)	
Globalization			−0.000958626 (0.00143931)			0.00528695 (0.0113695)
Ln GDP per capita	0.269481 *** (0.00748153)	0.281922 *** (0.00534777)	0.298147 *** (0.0121872)	0.213976 *** (0.0657183)	0.379137 *** (0.0855967)	0.290284 ** (0.136305)
GDP growth	0.00088624 (0.00219032)	0.000487233 (0.00200030)	0.00137128 (0.00202637)	0.0104900 (0.0102529)	0.0160616 (0.0107119)	0.104712 (0.0304211)
Ln Arable land	0.113109 *** (0.00647071)	0.0888971 *** (0.00604065)	0.0952195 *** (0.00531122)	0.210299 *** (0.0701103)	0.158903 *** (0.0597781)	0.156196 *** (0.0598957)
Ln Agricultural production	−0.0204668 *** (0.00516753)	−0.0144307 ** (0.00595875)	−0.0180365 *** (0.00579867)	0.0788502 ** (0.0385403)	0.0956332 ** (0.0416021)	0.0896892 ** (0.0396425)
Rural Population	0.00766082 *** (0.000398240)	0.00742802 *** (0.000503302)	0.00766486 *** (0.000500103)	0.0131988 *** (0.00343404)	0.0137986 *** (0.00355062)	0.0138899 *** (0.00351928)
Ln Population growth	−0.00294469 (0.00394025)	−0.00180546 (0.00359173)	−0.00586717 * (0.00349110)	0.0664214 (0.0460942)	0.0626977 (0.0444805)	0.476948 (0.103866)
Natural Disaster	2.40071 (2.30896)	2.15373 (2.03266)	3.27440 (2.55524)	−6.90303 *** (2.64627)	−6.55824 *** (2.75441)	−6.48437 ** (2.74300)
Ln Inflation	0.0660458 *** (0.00784140)	0.0747960 *** (0.00900465)	0.0798373 *** (0.00939306)	−0.0112384 (0.0376728)	0.00051853 (0.0402414)	0.00852784 (0.0394147)
AR (1) errors test	−1.62376 (0.1044)	−1.60499 (0.1085)	−1.65661 (0.0976)	−3.61083 (0.0003)	−3.29418 (0.0010)	−3.59656 (0.0003)
AR (2) errors test	1.51318 (0.1302)	1.53077 (0.1258)	1.57954 (0.1142)	−0.890439 (0.3732)	−1.19194 (0.2333)	−0.890439 (0.3732)

Note: *, **, *** stands for 10%, 5%, and 1% significant level, respectively. Av. stands for average; Ln stands for natural logarithm; AR stands for autoregressive.

## Appendix A

See Table A1.

Table A1. List of European countries used in the analysis (33), because the data are available for this countries.

**Table A1:** List of European countries used in the analysis.

Albania	Belarus	Belgium	Bosnia and Herzegovina
Bulgaria	Croatia	Cyprus	Czechia
Denmark	Estonia	Finland	France
Germany	Greece	Ireland	Italy
Latvia	Luxembourg	Netherlands	Norway
Poland	Portugal	Republic of Moldova	Romania
Russian Federation	Slovakia	Slovenia	Spain
Sweden	Switzerland	Turkey	Ukraine
United Kingdom			
